# A3SOM, abstained explainable semi-supervised neural network based on self-organizing map

**DOI:** 10.1371/journal.pone.0286137

**Published:** 2023-05-25

**Authors:** Constance Creux, Farida Zehraoui, Blaise Hanczar, Fariza Tahi

**Affiliations:** Univ Evry, IBISC, Université Paris-Saclay, Evry-Courcouronnes, France; Guru Ghasidas Vishwavidyalaya: Guru Ghasidas University, INDIA

## Abstract

In the sea of data generated daily, unlabeled samples greatly outnumber labeled ones. This is due to the fact that, in many application areas, labels are scarce or hard to obtain. In addition, unlabeled samples might belong to new classes that are not available in the label set associated with data. In this context, we propose A3SOM, an abstained explainable semi-supervised neural network that associates a self-organizing map to dense layers in order to classify samples. Abstained classification enables the detection of new classes and class overlaps. The use of a self-organizing map in A3SOM allows integrated visualization and makes the model explainable. Along with describing our approach, this paper shows that the method is competitive with other classifiers and demonstrates the benefits of including abstention rules. A use case is presented on breast cancer subtype classification and discovery to show the relevance of our method in real-world medical problems.

## 1 Introduction

Technological advances and an increase in the capabilities of modern computers in recent years have led to the production of massive amounts of data. This is true for many domains, such as the biomedical field, where images, genomic data, and other types of complex data are created faster than they can be processed by humans. As a response, applications of artificial intelligence—and deep learning, specifically—have become commonplace in these fields [1–6]. A significant part of this data remains unlabeled, i.e., it has not been assigned a label, as extracting information from it is challenging. Unsupervised learning, especially clustering, is typically used as a first step to explore data by grouping samples that exhibit similar patterns. In this work, we are interested in classification problems for which supervised learning methods are generally used. However, some real-world applications are characterized by the availability of only a few labels associated with certain samples. In this case, adding the information carried by unlabeled samples can improve separation between classes, leading to better classification results. The missing labels problem can be decomposed into two different subproblems. The first one, the most common, happens when some training examples have no labels; however, all classes are represented in the training set. Semi-supervised learning mainly focuses on this problem. The second subproblem is relative to the absence of labeled examples of some classes in the training set. For instance in oncology, cancer with the same primary site can have multiple subtypes associated with various clinical outcomes. As we study more and more patients, we might discover a new group of patients that have a new cancer subtype. In addition, another problem with classification is that some samples may be located in areas of overlap between classes. To deal with the last two issues, we can use abstained classification, also known as reject or selective classification. It enables the model not to classify observations if prediction confidence is too low. Abstention, when combined with semi-supervised classification, can help discover new classes in the data.

In the past decade, interest for explainable artificial intelligence models has rocketed. This move away from so-called ‘black-box’ models encourages developers to create models that can be understood by humans. In that regard, self-organizing maps (SOM) are a particularly interesting type of neural network. It learns to locate the position of training samples onto a topographic map of neurons. These neurons are prototypes that represent the data, and can be used to offer a prototype-based explanation. Moreover, the map is bi-dimensional and can be visualized.

In this context, we propose A3SOM, an Abstained explainable Semi-Supervised neural network based on a Self-Organizing Map. Dense layers are linked to the SOM to exploit available labels and associate meaning with the SOM neurons. The architecture of our method makes it explainable through the local arrangement of neurons, their visualization, as well as the use of two distinct abstention rules. Our model is able to perform two tasks:

- Standard semi-supervised classification, which leverages both unlabeled and labeled data complemented with visualization and explainability.
- Abstained semi-supervised classification, which allows the detection of classification ambiguities and the discovery of new classes.

This paper is organized as follows. We first present related works on semi-supervised classification, abstained classification, and extensions of SOM. We then detail our approach before presenting experimental results for the two tasks that A3SOM can perform, and show an application on breast cancer subtype classification. Finally, we conclude and present perspectives for future work.

## 2 Related works

This section presents the key points identified in the introduction: semi-supervised learning and abstained classification. As the SOM is an integral part of our algorithm, we also present extensions made to this model in recent years, including semi-supervised SOM and abstained SOM.

### 2.1 Semi-supervised learning

Semi-supervised learning is a middle ground between supervised and unsupervised methods: it uses labels when available, but unlabeled samples are not discarded, and are instead used in various ways to improve performance. Its use is particularly relevant when unlabeled data outnumbers labeled data [7]. Over the years, many semi-supervised methods have been developed, especially for image classification tasks [8–11].

In [12], multiple types of semi-supervised classification methods are described, with different ways of integrating unlabeled samples: *wrapper methods*, *unsupervised preprocessing*, *intrinsically semi-supervised methods*, and *transductive methods*.

**Wrapper methods** are based on classifiers. In the case of self-training [13, 14], a classifier is trained on labeled samples, and a class is predicted for all samples, including unlabeled ones. Predictions with high confidence are added to the set of training labels, and the process is repeated. Wrapper methods also include approaches based on boosting [15] or co-training [16].

**Unsupervised preprocessing** is when computations are performed on unlabeled data to make training on labeled data more efficient. Feature extraction can be done on unlabeled data, by transforming the original dataset to a new one with fewer features, for example with AutoEncoders [17, 18]. Clustering can also be used to represent the unlabeled data in groups before classification [19]. Preprocessing can also refer to pre-training [20, 21], meaning that weights of the model are first learned on unsupervised data, and they only need to be adjusted (fine-tuned) with labeled data to fit the classification problem better.

**Intrinsically semi-supervised methods** include both labeled and unlabeled data in the error computation. For example, this can be done by adding a regularization term based on unsupervised samples in the loss function [22–24], or by adapting existing architectures such as GAN (Generative Adversarial Networks) by associating one output to fake data points [25].

**Transductive methods** are typically graph-based methods, where label information is propagated along edges [26]. One commonly used method is label propagation [27], which uses neighboring data to predict the labels of unlabeled samples.

### 2.2 Abstained classification

In medical diagnosis and other fields like fraud detection or self-driving vehicles, wrongly classifying a sample can have critical consequences. Abstention, also called rejection, is a branch of classification that enables the model to abstain from returning predictions when confidence is too low. Abstained classification is primarily associated with supervised learning models. Semi-supervised applications are limited to very particular data types presenting spatial relationships that can be exploited for abstention [28–32]. Different abstention criteria can be defined using thresholds on provided predictions.

#### 2.2.1. Abstention criteria

It is possible to differentiate between two rules to explain why abstention is applied to a prediction: the distance rule and the ambiguity rule. In [33, 34], both types are described in the context of neural networks with a sigmoidal output. The *distance* (or novelty) rule identifies samples that do not seem to belong to any known class. A prediction is abstained from if the highest predicted probability for a sample is lower than a threshold. This concept is similar to open-set classification problems [35–38], which account for situations where test data might not have the same distribution as training data.

#### 2.2.2. Thresholds

Applying thresholds on outputted predictions is common. In a pioneer work on rejection [39], Chow defines a rule with a global rejection threshold: the model abstains from classifying an input sample if the probability that it belongs to a class is lower than a predefined threshold that depends on abstention cost. This approach assumes complete knowledge of the a priori probability distribution of classes and the a posteriori probabilities, which is rarely the case in real-world problems. In [40], authors propose to use one threshold for each class, i.e., local thresholds, instead of one global threshold for all classes. They show that local thresholds lead to better results when class probabilities are estimated.

The *ambiguity* rule detects instances where two classes overlap, and the threshold is applied to the difference between the two highest predicted probabilities.

### 2.3 Extensions of SOM

Self-organizing maps were first described as an unsupervised neural network by Kohonen in [41], and present unique properties for clustering and interpretability. Many extensions of the SOM model have been proposed, often extending SOM to supervised learning [42, 43], by including the label as one of the features [44, 45] or by labeling the SOM neurons a posteriori, typically with majority voting [46, 47].

#### 2.3.1 Semi-supervised SOM

SOM have seldom been integrated into semi-supervised contexts. Existing works could primarily be categorized as *intrinsically semi-supervised*, as unlabeled and labeled samples are processed in the same step to optimize the model. It is done by computing an error using unlabeled and labeled information to modify SOM neurons or add new ones [48–52]. Adding neurons to SOM can create clusters more relevant to real-life organization but has the disadvantage of deforming the map, making its visualization less straightforward. *Unsupervised preprocessing* is also employed with SOM, by training a first unsupervised SOM before using it in concordance with label information [53]. As SOM present a meaningful topology, *transductive methods* such as label propagation can also be used [54].

#### 2.3.2 Abstained SOM

To our knowledge, only four works associate abstention with SOM, all for supervised classification, which can be cited. ROSOM [55] trains a SOM, then the neurons are labeled with a majority vote. According to a rejection threshold, some neurons are instead associated with rejection. Another method uses a combination of SOM and the traveling salesman algorithm to reject the classification of outliers [56]. Both of these works detect what we call distance abstention. IRSOM [57] uses a SOM in a binary classification problem to detect ambiguities. Finally, SLSOM [58] trains an unsupervised SOM followed by a dense layer to perform classification. This method separates ambiguity and distance rejection, and uses global thresholds.

*2.3.2.1 Identified gaps in related works.* There is a lack of methods combining semi-supervised learning and abstained classification. To our knowledge, only a few methods integrate both concepts: almost all of them can only be applied to images [28–31], except for one that is suited for graphs [32]. In this new work, we propose an intrinsically semi-supervised method by including the SOM in the regularization process. This method can be used on tabular data. When associated with local abstention options, our approach enables the discovery of new classes and the explanation of predictions.

## 3 Method

**Fig 1:**
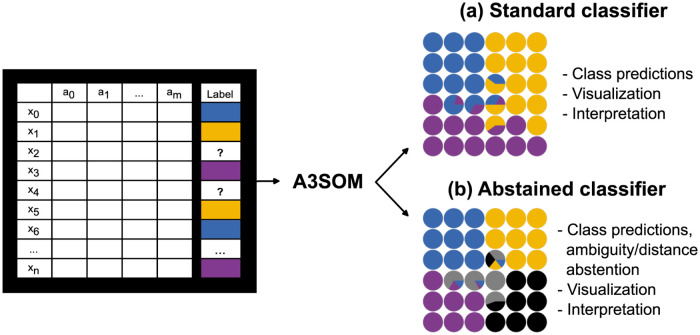
Overview of A3SOM method. Input data is tabular, there can be missing labels. A3SOM can perform standard classification or abstained classification. In both cases, results can be visualized and interpreted.

We present a novel semi-supervised learning approach called A3SOM. It is based on self-organizing maps and therefore includes data visualization. Fig 1 shows the inputs and outputs of A3SOM, illustrating the two tasks it can perform: standard or abstained classification.

Data is first given as input to a SOM, which clusters the samples and provides data visualization. The output of the SOM is then fed to a block of dense layers, which produces the output. Unlike standard SOM that are based on unsupervised learning, we use a SOM in a semi-supervised learning process where the positions of SOM units are not only influenced by the distance between instances (unlabeled and labeled) and neuron prototypes, but also by the labels used in the training step. There are two options for prediction. The first is a standard classification task with visualization and interpretation (Fig 1a), and the second consists in an abstained classification task capable of detecting ambiguities and discovering new classes through the use of the SOM and analyzing the results (Fig 1b).

In this section, we first present the full A3SOM learning algorithm. We then show how our method performs standard classification, and how we can explain the results. We finally present how our approach can be used for abstained classification where new classes can be discovered and ambiguities between existing classes can be detected.

### 3.1 A3SOM training phase

**Fig 2:**
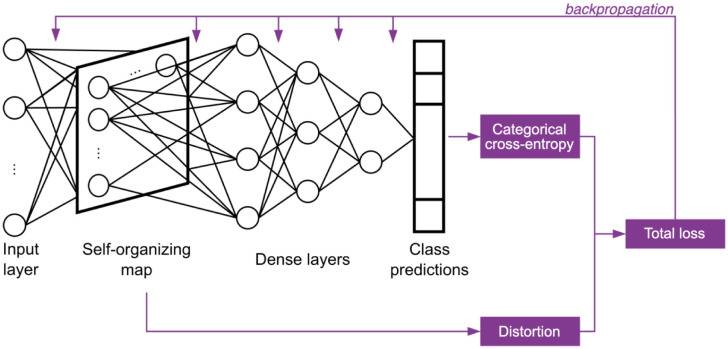
A3SOM training phase. Information is propagated from the input layer to the self-organizing map and to fully-connected dense layers, to obtain class predictions. A loss composed of the categorical cross-entropy and the distortion of the SOM is computed. Then, the weights of the model are updated during backpropagation.

Let $X=\{X_{U},X_{L}\}$ be the set of samples where $X_{U}$ are the unlabeled input samples and $X_{L}$ the labeled ones. $Y_{L}$ are the true labels associated to $X_{L}$. The set of predicted labels is defined as $\hat{Y}=\left\{\hat{Y_{U}},\hat{Y_{L}}\right\}$, where $\hat{Y_{U}}$ and $\hat{Y_{L}}$ are associated respectively with $X_{U}$ and $X_{L}$. *N* is the total number of samples in *X*, and $N_{L}$ is the number of labeled samples. The training of the network is carried out in two dependent phases: forward propagation and backpropagation. During the forward propagation step, data is propagated through the different layers of the network, as shown in Fig 2, from the input layer to the last layer of the dense block. Class prediction is then performed, and an error is calculated using the total loss function, which is composed of two main terms: the distortion and the categorical cross-entropy. Afterward, gradient descent is used during the backpropagation step to update the model parameters in the different layers of the network, depending on the error. In this step, parameters are modified from the last dense layer to the input layer.

#### 3.1.1 Forward propagation

*3.1.1.1 Self-organizing map.* The matrix *X* is given to the SOM as input. The map is composed of neurons arranged on a two-dimensional grid. Each neuron unit *u* in the SOM is associated to two vectors: a vector $r_{u}=\left(r_{u}^{1},r_{u}^{2}\right)$ that represents the coordinates of *u* in the grid and a weight vector $w_{u}={\left[w_{u}^{1},w_{u}^{2},...,w_{u}^{m}\right]}^{T}\in R^{m}$, which has the same dimension as the input vector *x*_*i*_ ∈ *X* where $x_{i}\in R^{m}$, *m* being the number of features in the original data space. Each neuron unit corresponds to a cluster, and the weight vector associated with the unit is the prototype that represents the cluster.

The first step of the SOM algorithm is to identify the neuron closest to each sample. These closest neurons, called Best-Matching Units (BMUs) or winner neurons, are found by measuring a distance, for example the Euclidean distance, between the sample and the SOM prototypes. The BMU of a sample *x*_*i*_ is the neuron with the smallest distance to *x*_*i*_:

$$\begin{array}{c} BMU\left(x_{i}\right)=\text{arg}\;\underset{u\in U}{\text{min}}\;d(x_{i},w_{u}) \end{array}$$ (1)

where *U* is the set of neurons and *d*() the distance measure.

In the original SOM model, prototypes *w*_*u*_(*t*) of the BMU and its neighbors are updated towards the sample *x*_*i*_:

$$\begin{array}{c} w_{u}\left(t+1\right)=w_{u}\left(t\right)+\alpha \left(t\right)H_{t}\left(BMU\left(x_{i}\right),u\right)\left[x_{i}-w_{u}\left(t\right)\right] \end{array}$$ (2)

where *α*(*t*) is the learning rate set by the optimizer and the neighborhood function is $H_{t}\left(BMU\left(x_{i}\right),u\right)=exp\left(-\frac{d^{\prime }{\left(r_{BMU(x_{i})},r_{u}\right)}^{2}}{2\sigma^{2}\left(t\right)}\right)$. *σ*(*t*) is the neighborhood function’s radius, and *d*′() is the distance between the coordinate vectors of the units in the map, which can be different from the previously defined d().

In this method, we use a variant of SOM weight update, in which weights are updated after seeing mini-batches of data. It consists of two steps: a fixed assignment step, after which weights are updated by minimizing a measure of the SOM error (defined in Eq 8). The assignment step consists in finding the BMU for each sample in the current batch of data. Then, the following formula is used to update prototype weights:

$$\begin{array}{c} w_{u}\left(t+1\right)=\frac{\sum_{v\in U}H_{t}\left(u,v\right)\sum_{i=1}^{N}Ind\left(x_{i},v\right)*x_{i}}{\sum_{v\in U}H_{t}\left(u,v\right)\sum_{i=1}^{N}Ind\left(x_{i},v\right)} \end{array}$$ (3)

where *Ind*(*x*_*i*_, *v*) is the indicator matrix, in which all entries are equal to 0 except for those where the BMU of sample *x*_*i*_ is the neuron *v*, where it is equal to 1.

The activation of a neuron *u* ∈ *U* corresponds to the distance between data samples and the prototype associated with the neuron. It is transmitted to the dense block and can be computed as follows:

$$\begin{array}{c} a_{u}=d\left(x,w_{u}\right) \end{array}$$ (4)

*3.1.1.2 Dense block.* The dense block consists of *B* fully-connected dense layers, *B* being a hyperparameter of the model. The first layer receives the output of the SOM as input. The activation of the neuron *l* in the hidden layer *j* (1 ≤ *j* ≤ *B*) is defined as:

$$\begin{array}{c} h_{l}^{j}=f(\sum_{k}w_{kl}^{j}\;h_{k}^{j-1}+b_{l}^{j}) \end{array}$$ (5)

where $w_{kl}^{j}$ is the weight of the connection linking the neuron *l* of the layer *j* to the neuron *k* of the layer (*j* − 1), $b_{l}^{j}$ is the bias of the neuron *l* in the layer *j* and $h_{k}^{j-1}$ the activation of the neuron *k* in the layer (*j* − 1) ($h_{k}^{0}=a_{u_{k}}$ with $a_{u_{k}}$ the activation of the unit *u*_*k*_ in the SOM). *f*() is the activation function: specifically, we use the Rectified Linear Unit (ReLU) function, where ReLU(x) = max(0,x).

The last dense layer is composed of as many neurons as there are classes in the training set. The output of a neuron *c* in the last layer is obtained by:

$$\begin{array}{c} y_{c}=f^{out}(\sum_{k}w_{kc}^{out}\;h_{k}^{B}+b_{c}^{out}) \end{array}$$ (6)

where $w_{kc}^{out}$ and $b_{c}^{out}$ are respectively the weights and the biases of the output layer and $h_{k}^{B}$ the activation of the last hidden layer, *k* is the index of the neuron and *c* the index of the class. After the last layer, the class-membership probabilities for all the samples in *X* can be obtained using an activation function *f*^*out*^(). We will use the softmax or the sigmoid activation function depending on the task performed.

#### 3.1.2 Backpropagation

Gradient descent is used to update the weights of the network neurons, in both the SOM and the dense block. The semi-supervised loss is composed of two terms.

- The first is a supervised term, the cross-entropy cost function, which is defined as:
  $$\begin{array}{c} CE\left(Y_{L},{\hat{Y}}_{L}\right)=-\sum_{i=1}^{N_{L}}\sum_{c=1}^{C}y_{ic}\text{ln}{\hat{y}}_{ic} \end{array}$$ (7)
  where *C* is the number of classes, ${\hat{y}}_{ic}$ is the output of a neuron *c* (corresponding to the class *c*) in the last layer for a sample *x*_*i*_ and *y*_*ic*_ is the *c*^*th*^ component of the vector *y*_*i*_ representing the true label associated to the sample *x*_*i*_.
  Note that *y*_*i*_, for $1\leq i\leq N_{L}$, is represented as a one-hot encoded vector of dimension *C*, i.e. *y*_*i*_ = (*y*_*i*1_, …, *y*_*iC*_) with *y*_*ic*_ = 1 if *y*_*i*_ corresponds to the class *c* and *y*_*ic*_ = 0 otherwise.
- The second term corresponds to the distortion. Assessing the quality of the SOM is more complex than simply using quantitative criteria like for most clustering validation problems [59]: we also want to know whether the map preserves topology relationships, i.e., if neighboring samples in the original data are also neighbors on the map. Distortion is the cost function of SOM that comprises both quantization and topology errors. It measures the average error made when projecting data from its original space onto the SOM, weighted by the neighborhood function. It is defined by:
  $$\begin{array}{c} D\left(X,U\right)=\frac{1}{N}\sum_{i=1}^{N}\sum_{u\in U}H_{t}\left(BMU\left(x_{i}\right),u\right)*d{\left(x_{i},w_{u}\right)}^{2} \end{array}$$ (8)
  where *x*_*i*_ ∈ *X*.

In a semi-supervised context, labels may not be available for all samples. This means that some samples cannot be used in the calculation of the cross-entropy. However, these unlabeled samples are integrated into the calculation of the distortion. The loss function can be formulated as follows:

$$\begin{array}{c} L\left(X,U,Y,\hat{Y}\right)=CE\left(Y_{L},\hat{Y_{L}}\right)+\gamma D\left(X,U\right)+{\eta ||w||}_{2} \end{array}$$ (9)

where ||*w*||_2_ is a regularization term, and *γ* and *η* are the parameters that control the importance of the distortion and the regularization term in the loss.

### 3.2 A3SOM prediction phase

Compared to the majority of semi-supervised approaches, the originality of our approach lies in the integration of the SOM inside the network architecture, allowing analysis and interpretation of the results. Indeed, the location of the SOM neurons is influenced by the existing labels and the distances between all data and SOM weight vectors. Below are presented the prediction phases corresponding to the standard classification task and the abstained classification task.

#### 3.2.1 A3SOM for classification task

After the last layer, the class-membership probabilities for all the samples in *X* can be obtained by using the softmax activation function. Here, it is an appropriate choice of activation function: we are in a multi-class classification problem, where we consider each class to be mutually exclusive. In this case, the equation defined in Eq 6 can be re-written as:

$$\begin{array}{c} y_{ic}=f^{out}\left(z_{i}\right)=softmax\left(z_{i}\right)=\frac{exp(z_{i})}{\sum_{j}exp\left(z_{j}\right)}. \end{array}$$ (10)

**Fig 3:**
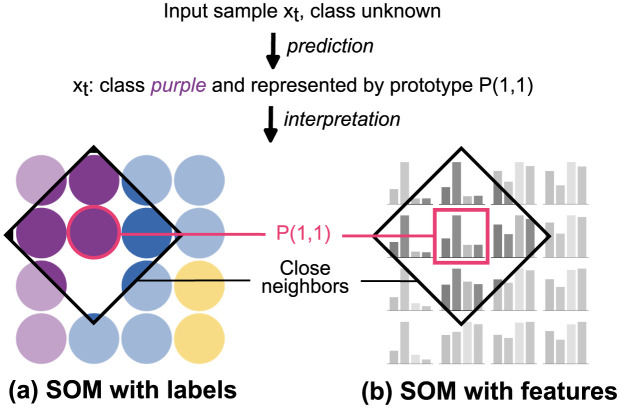
Interpretation of prediction. The model predicts the class of an input sample *x*_*t*_ as *purple*. This sample is represented by the prototype P(1,1) which can be visualized.

Thanks to the use of a SOM in our model, additional outputs can be obtained along with class predictions. This additional information can be visualized and makes the predictions explainable. We can return the BMU prototype of each sample and analyze the properties of the samples contained in the cluster represented by the BMU. This type of interpretation is called a prototype-based explanation [60]. In addition, we can study the BMU’s neighbors since neurons that are close on the map share similar semantic concepts. The labels associated with the neurons can also be analyzed in order to understand if the sample is close to neurons that are all associated with the same class or not. An illustration of prediction explanation is given in Fig 3, where A3SOM predicts an input sample *x*_*t*_ from the test set. A class prediction is returned, as well as a prototype-based explanation associated with different visualizations.

In Fig 3a, the SOM neurons are colored based on the labels of the samples they represent. We see that all of the samples represented by P(1,1) are from the same class as *x*_*t*_. *x*_*t*_ is also close to P(1,2), which means it has similarities with class *blue*. In Fig 3b, the SOM neurons are represented by their values for each of the dataset’s features. The feature profile of P(1,1) is similar to the profile of other prototypes of class *purple*, with high values for the second feature, and lower values for other features. P(1,1) is the prototype from class *purple* with the highest values for the last two features, which typically have high values in class *blue*, which it is close to with P(1,2).

#### 3.2.2 A3SOM for abstained classification task

**Fig 4:**
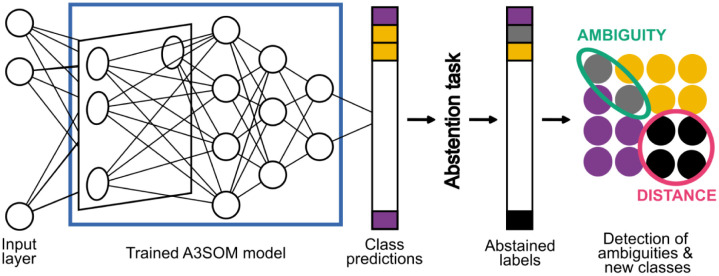
Abstained classification task overview. The abstention task is applied after the model has been trained. It produces a new set of labels that can be visualized.

In order to analyze ambiguities between existing classes and discover new classes, we propose an extension of the model to perform abstained classification, illustrated in Fig 4. An abstention task is plugged to the classifier’s output, and the decision is based on the estimation of the posterior class probabilities.

We use the two abstention rules described in Section 2.2. The first is the distance rule, which abstains from predicting samples that are far from learned classes and potentially belong to new classes. The second is the ambiguity rule, which abstains from predicting samples that are in ambiguous classification areas (where several classes are overlapping). We make the assumption that a sample can only belong to one class. Both rules depend on the output of the neurons located in the output layer, *y*_*ic*_, which corresponds to the posterior probability *P*(*c*|*x*_*i*_) of an input *x*_*i*_ to be an element of the class *c*.

In order to distinguish the two types of abstention, the output is defined using a sigmoid activation function. Here, the equation defined in Eq 6 can be re-written as:

$$\begin{array}{c} y_{ic}=f^{out}\left(z_{i}\right)=sigmoid\left(z_{i}\right)=\frac{1}{1+\text{exp}(-z_{i})} \end{array}$$ (11)

Indeed, while the softmax function is appropriate for standard classification, its use in abstained classification is unsuitable. A softmax function forces all probabilities to sum to one, which is undesirable in a context where a sample is far from all known classes, or located in the overlap between several classes. Since probabilities returned for each class by the sigmoid function are independent, it is more appropriate for abstained classification problems.

The thresholds for abstention can be defined globally (the same threshold for all classes) or locally (one threshold per class). A3SOM uses local thresholds as they are more flexible and better suited to obtain optimal decisions and abstention regions. Thus, in the definitions below, one threshold *β*_*c*_ is defined for each predicted class *c*.

*3.2.2.1 Distance abstention rule.* In the distance rule, we abstain from returning a prediction if the largest output *y*_ic*_ is lower than a threshold $\beta_{dist}^{c^{*}}$. Thus we can define the distance abstention rule *rule*_*dist*_ by:

$$\begin{array}{c} rule_{dist}\left(x_{i}\right)=\{\begin{array}{cc} 1 & \text{if}\;y_{ic^{*}}<\beta_{dist}^{c^{*}}\\ 0 & \text{otherwise} \end{array} \end{array}$$ (12)

where *y*_ic*_ = max_*c*_{*y*_*ic*_} and $0\leq \beta_{dist}^{c^{*}}\leq 1$.

A low distance threshold will lead to few abstentions because the best probability is rarely too low. It is when the threshold increases that the distance rule becomes more stringent.

*3.2.2.2 Ambiguity abstention rule.* In the ambiguity rule, we abstain from predicting the input *x*_*i*_ if the difference between the two largest output probabilities *y*_ic*_ and *y*_ic**_ (*y*_ic*_ = max_*c*_{*y*_*ic*_} and *y*_ic**_ = max_*c* ≠ c*_{*y*_*ic*_}) is lower than a threshold $\beta_{amb}^{c^{*}}$. We can define the ambiguity abstention rule *rule*_*amb*_ by:

$$\begin{array}{c} rule_{amb}\left(x_{i}\right)=\{\begin{array}{cc} 1 & \text{if}\;y_{ic^{*}}-y_{ic^{**}}<\beta_{amb}^{c^{*}}\\ 0 & \text{otherwise} \end{array} \end{array}$$ (13)

where $0\leq \beta_{amb}^{c^{*}}\leq 1$.

A small ambiguity threshold means we will only abstain from prediction when the two highest probabilities are very close. The higher the ambiguity threshold, the more we abstain from predictions.

## 4 Results

A3SOM is implemented using the TensorFlow [61] Python API, Keras [62], and the implementation of a Keras layer for SOM given in [63].

We present results for each task performed by A3SOM. For standard semi-supervised classification, we illustrate A3SOM’s performance compared to other classifiers on several datasets (Section 4.1). For the abstained classification task, we show using an artificial dataset that abstaining from prediction can improve accuracy and reduce the number of classification errors (Section 4.2). Finally, we illustrate the usefulness of A3SOM in a case study relating to the diagnosis of breast cancer (Section 4.3).

### 4.1 Semi-supervised classification evaluation

In this section we compare A3SOM’s semi-supervised classification results to other methods on several datasets.

#### 4.1.1 Datasets

We use a total of six datasets in the benchmark. The first is an artificial dataset we generated to show the specificities of our method. The others are five public datasets.

**Fig 5:**
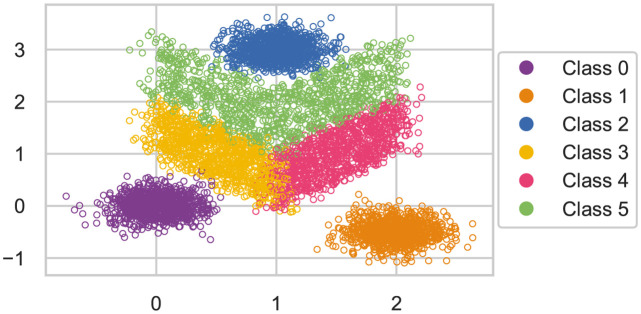
Plot representing the 2D points of the artificial dataset. Each class in the dataset is represented by a different color. Some overlaps between classes can be noticed.

The artificial dataset is composed of 6 classes: three defined by Gaussian clusters (labeled 0, 1 and 2) and three with uniform distributions (labeled 3, 4 and 5), as shown in Fig 5. The classes are generated in order to have different degrees of ambiguity. Classes 3, 4 and 5 have a high ambiguity in their overlapping areas; class 5 overlaps with class 2; classes 0 and 3 are close but still separable, and class 1 is well separated from others. Dataset construction details are given in supplementary file A, Section 1.

The real-world datasets are extracted from the OpenML database [64]:

- Cardiotocography: measurements data on cardiotocograms [65].
- Ionosphere: radar data [66].
- Iris: different types of flowers [67].
- MNIST: handwritten digits [68].
- WDBC: breast lump description [69].

All datasets are all normalized between 0 and 1 to reduce measurement bias, and instances with missing values are removed. A summary of the six datasets used is given in Table 1.

**Table 1:** Description of the different characteristics of benchmark datasets: Size, number of features, and number of classes. Note that some datasets are not balanced.

	Instances	Features	Classes	Balanced
**Artificial**	3 000	2	6	Yes
**Cardiotocography** [65]	2 126	23	3	No
**Ionosphere** [66]	351	34	2	No
**Iris** [67]	150	4	3	Yes
**MNIST** [68]	70 000	784	10	Yes
**WDBC** [69]	683	10	2	No

#### 4.1.2 Benchmark methods

We compare A3SOM to other semi-supervised methods, which we can divide into three categories: implemented baselines, and existing semi-supervised approaches not SOM-based (SSL approaches) or SOM-based (SOM approaches).

*4.1.2.1 .Implemented baselines.* We implemented three combinations of self-training [13], which is a *wrapper method*, with popular classifiers: Support-Vector Machine (SVM) [70], Random Forest (RF) [71], and Multi-Layer Perceptron (MLP) [72]. Only the last is a deep learning algorithm. The package scikit-learn [73] is used for implementation. Self-training is when labeled data is used to build a first classifier, which predicts ‘pseudo-labels’ for unlabeled samples. Pseudo-labels are then used along with labels for rounds of training. Label propagation (LP) [27] is also implemented with scikit-learn. This is a *transductive method* that builds a fully-connected similarity graph over the training samples, and label information is propagated to unlabeled samples in the graph.

*4.1.2.2 SSL approaches.* Few semi-supervised classification approaches can be applied on tabular data. We include two recent methods for which code was made available: VIME [24] and TabNet [21]. VIME is designed to extend the use of self- and semi-supervised learning to tabular data. It uses an encoder to learn a new representation of the data focusing on important features. It is *intrinsically semi-supervised*, combining a supervised and an unsupervised term in the loss. TabNet [21] is an attention-based model that learns which features are important for prediction. Through *unsupervised preprocessing* and supervised fine-tuning, TabNet can be used as a semi-supervised method.

*4.1.2.3 SOM approaches.* For semi-supervised methods based on SOM, we include the only recent methods which made their code available, SS-SOM [51] and SuSi [53]. SS-SOM is based on a growing SOM which switches between supervised and unsupervised learning depending on the nature of each sample. If the sample is unlabeled, the standard unsupervised SOM algorithm is performed. Otherwise, the label will influence the decision when the closest neuron is found: the label associated with the neuron might change, or a new neuron might be inserted into the map. The method can be seen as *intrinsically semi-supervised*. Specifically, we use the variant Batch SS-SOM (BSSSOM) [51] as it is the only one with an explained python package [74] that can be included in our experimental protocol. SuSi [53] first trains an unsupervised SOM on all data, then uses a second SOM to associate labels to the neurons of the first map. It is a method using *unsupervised preprocessing*. The accompanying package [75] is used. Note that this method’s computations on MNIST were extremely long, thus they are not presented here.

A comparison of our method with implemented supervised classifiers can be found in supplementary file A, Section 2, as well as details on the hyperparameters used for the methods presented above.

#### 4.1.3 Benchmark results

A3SOM was compared to the methods presented in the previous section. We defined different levels of labeled data seen during the training phase as percentages of the total training data available. The labels of the remaining samples were set to -1. Thus, all semi-supervised methods were trained on the entire dataset, where only some of the samples were labeled. All methods were evaluated on the same validation sets, in which all labels were present. For each method, different sets of hyperparameters were implemented. We present the variant with the highest score for each percentage of labeled data. The methods were scored on their mean validation accuracy when performing five-fold cross-validation.

**Fig 6:**
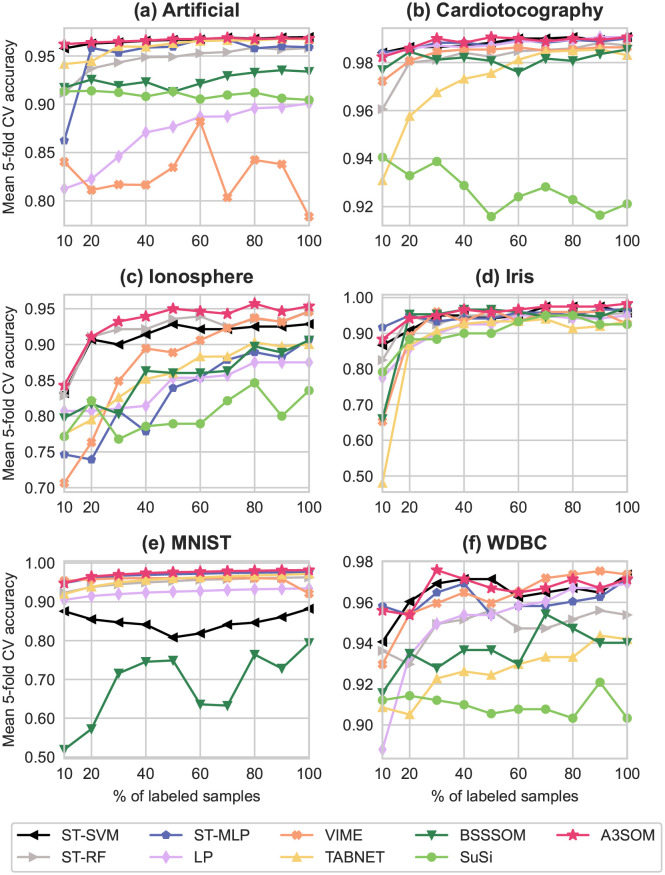
Benchmark results. Representation of the mean validation accuracy after 5-fold cross-validation for different percentages of labeled data used during training. The x-axis represents the percentage of labeled samples included during training, and the mean accuracy of each method can be read on the y-axis.

Fig 6 gives the results obtained by A3SOM and the other classifiers on the datasets described in Section 4. The numerical values of the results, along with the standard deviation, are given in supplementary file A, Section 3.

As we can see on the figure, a general trend is that the accuracy of the different methods improves as more labeled data is included during training, which is predictable.

*4.1.3.1 Implemented baselines.* LP, ST-RF and ST-SVM are not deep-learning methods; we expect them to perform well on smaller datasets, and less so on bigger datasets. LP behaves as anticipated. ST-RF actually performs well on most datasets except for Artificial and WDBC, for which scores are slightly lower. ST-SVM also reaches a good accuracy for the most part, with one notable exception on MNIST, where results are low; moreover, it was extremely slow to run. ST-MLP generally makes good predictions, although its performance is relatively low on Ionosphere.

*4.1.3.2. SSL approaches.* VIME and TabNet both obtain good accuracies for MNIST and Iris. VIME performs well on datasets Cardiotocography and WDBC; and TabNet on the Artificial dataset. On the other datasets, however, both methods are slower to increase and do not always catch up, even with 100% of labeled data. This is the case with Artificial for VIME, and with WDBC for TabNet.

*4.1.3.3 SOM approaches.* SuSi seems to struggle on bigger datasets, and although BSSSOM obtains high scores on Iris and Cardiotocography, its performance is average on the other datasets.

*4.1.3.4 A3SOM.* We observe that A3SOM is among the most accurate classifiers in our benchmark, and gives the best results for all datasets except for WDBC. On the latter dataset, A3SOM obtains slightly lower accuracies than ST-SVM or VIME for some labeled cases. When considering the accuracies averaged on all percentages of labeled data, A3SOM gives the best results for all datasets, as shown in Table 2. Compared to the other methods, A3SOM’s competitiveness is coupled with additional advantages: visualization and explainability.

**Table 2:** Averaged benchmark accuracies. The results presented in Fig 6 are averaged over all percentages of labeled data. Best performance for each dataset is in bold.

	Artificial	Cardio.	Iono.	Iris	MNIST	WDBC
**ST-SVM**	0.966	0.988	0.91	0.945	0.848	0.965
**ST-RF**	0.947	0.982	0.92	0.935	0.951	0.948
**ST-MLP**	0.95	0.988	0.832	0.948	0.969	0.961
**LP**	0.87	0.988	0.843	0.912	0.925	0.95
**VIME**	0.827	0.984	0.875	0.915	0.956	0.963
**TABNET**	0.96	0.972	0.857	0.875	0.956	0.927
**BSSSOM**	0.925	0.981	0.856	0.928	0.686	0.936
**SuSi**	0.91	0.927	0.803	0.904	-	0.91
**A3SOM**	**0.967**	**0.989**	**0.932**	**0.957**	**0.973**	**0.967**

### 4.2 Abstained classification evaluation

We evaluate the performance of A3SOM’s abstained classification on the artificial dataset presented in Section 4.1.1. We first show the effect of global and local abstention thresholds on the improvement of classification accuracy. We then illustrate how extending the SOM with an abstained classifier enables the identification of samples within overlapping class boundaries and the discovery of new classes.

#### 4.2.1 Trade-off between performance improvement and abstention rate

We divide the classification outputs into two decision regions *G* and *E* such that *G* represents the good predictions and *E* represents the misclassified predictions (errors). Abstention allows us to divide the output space differently by defining two regions *A* and *R* so that *A* represents the accepted predictions and *R* the rejected ones. From these four regions, we can define two performance measures for abstained classification: Accepted Accuracy (AA) and Rejected Error (RE). AA corresponds to the correct prediction rate among the accepted predictions and RE to the rate of rejected errors among the total errors. They are defined as follows:

$$\begin{array}{c} \text{AA}=\frac{\left|G\cap A\right|}{\left|A\right|}\;\;\text{RE}=\frac{\left|E\cap R\right|}{\left|E\right|} \end{array}$$ (14)

**Fig 7:**
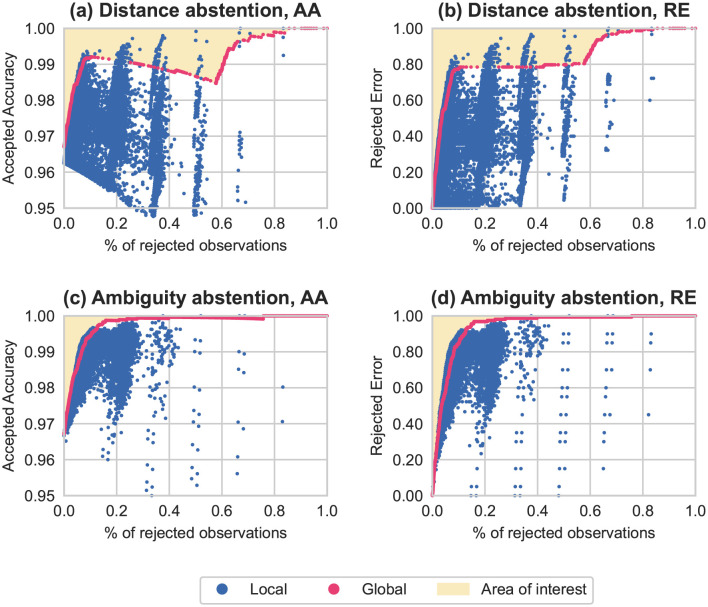
Evolution of performance on the artificial dataset when varying abstention thresholds. Scores obtained using combinations of local thresholds are in blue, and those obtained using global thresholds are in pink. The areas in yellow are the regions of interest, i.e., where local thresholds perform better than global ones. In **(a)** and **(b)** we vary distance abstention thresholds between 0 and 1 (ambiguity thresholds are set to 0). In **(c)** and **(d)** we vary ambiguity abstention thresholds between 0 and 1 (distance thresholds are set to 0).

Fig 7 shows how the model’s performances evolve when we vary the thresholds used for abstention. When all predictions are accepted (i.e., 0% of rejected observations on the figures), AA is equal to the standard classification accuracy, and RE is equal to zero as *E*∩*R* is empty.

In Fig 7 we see that, whether we apply distance or ambiguity abstention, AA and RE both rise with the number of abstentions. For a given AA or RE rate, there exist combinations of local thresholds that can outperform global thresholds while abstaining from fewer predictions. This is highlighted in the yellow areas. For example, on Fig 7b for distance abstention, it is necessary to reject a little more than 60% of the samples by global abstention to reach 90% of RE, whereas it suffices to abstain from about 20% of predictions to get the same score with local thresholds. This difference can be explained by the fact that proximity between the different classes varies, and classes have different organizations, hence the need to have specific abstention thresholds for each class.

The effect of varying the local threshold for individual classes is analyzed and given in the supplementary file B, Section 1.

#### 4.2.2 Interpretation of abstained classification

One advantage of this work is its ability to identify and visualize potential new classes and ambiguous classification areas in the input data using local abstention options. To show these properties, we split the artificial dataset into a training and a test set. The training set is curated to include data from all six classes that compose the dataset, but the labels for class 1 are removed and considered unknown. In this experiment, class 1 represents the new class that our algorithm must discover with the distance abstention rule. We also randomly removed 90% of labels across all the remaining classes, so that the hidden class is not the only one with masked labels.

For the distance rule, we found the highest predicted probability for each sample and associated it with the corresponding class. Distance thresholds were then set to the mean of all the probabilities associated with each class. For the ambiguity rule, we found the two highest probabilities for each sample, and associated the difference between the two with the class with the highest probability. Ambiguity thresholds were then set to the mean of all the differences associated with each class. Specific threshold values used for this application can be found in supplementary file B, Section 2.

**Fig 8:**
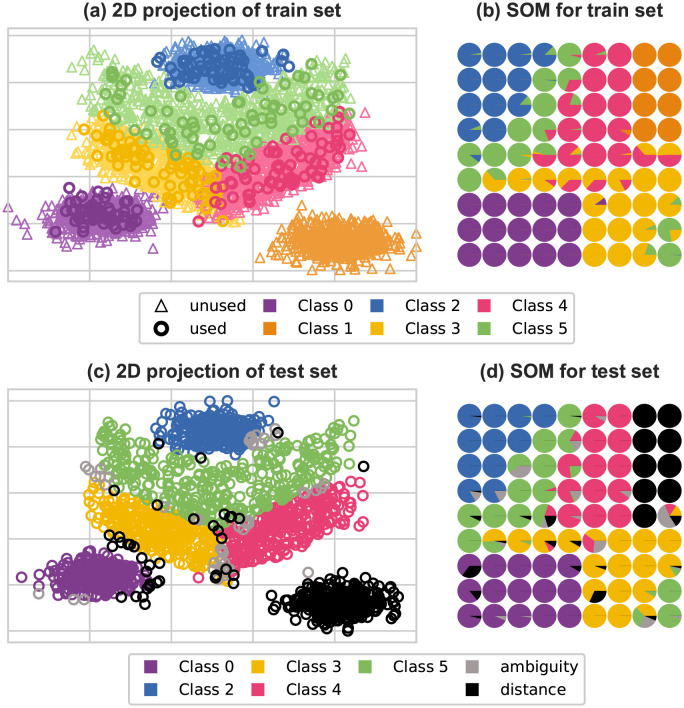
Representation of labels on the artificial dataset. **(a)** 2D representation of training samples, colored by their true labels. Triangles are for labels that were not used during training, and circles for labels used. **(b)** Representation of SOM prototypes by the true labels of the training set. **(c)** 2D representation of test samples, colored by their abstained label. **(d)** Representation of SOM prototypes by the abstained labels of the test set.

Fig 8 represents different sets of labels—before or after abstention—for the artificial dataset. In both cases, labels are shown on the projection of samples and on the SOM created by the model.

On Fig 8a and 8b re shown respectively the 2D points and the true label distribution in the SOM computed on the whole training set of the artificial dataset. In Fig 8a we can see the overlapping areas induced by classes 3, 4 and 5. We can also see how far class 1 is from the other ones. In this figure, the samples whose labels were seen during training are represented by circles, while the samples whose labels were masked during training are represented by lighter triangles.

In Fig 8b we show the true label repartition of the input data on the SOM. As expected, the input samples of classes 0, 1 and 2 are located at the corners of the map. On the contrary, input samples of classes 3, 4 and 5 are situated in the center of the SOM, where SOM neurons can be associated with samples from several classes, which shows the ambiguities identified in Fig 8a.

On Fig 8c and 8d are shown respectively the 2D points and the abstained labels repartition in the SOM computed on the test set. In Fig 8d we can see on the top right of the map that the model abstained from predicting a group of samples with the distance rule. The proximity of these examples in the SOM suggests they belong to the same class. We have verified that the abstained examples correspond to class 1 represented in Fig 8a. We can also see in Fig 8c that the model abstained from classifying input samples in ambiguous areas. For some of these predictions the ambiguity rule was applied, as expected. For others the distance abstention rule was applied. However, it is still apparent on the SOM that these samples do not form a group the way the samples in the top right do. Moreover, the abstention task did not find noticeable ambiguity around classes 0 and 2. This can be explained by the fact that classes 0 and 2 are separable.

### 4.3 Semi-supervised abstained classification: A case study

This section shows how A3SOM can be used on a real-life dataset to make essential observations or discoveries. Our example is in the field of medical diagnosis, using omics data. We demonstrate how our method can be used to classify breast cancer subtypes and discover a subtype that was not present in the training labels.

#### 4.3.1 Biological context

Breast cancer is the most diagnosed cancer in the world, as well as one of the most deadly. There are multiple types of breast cancer, called subtypes, which differ in genomic features and clinical outcomes. The four primary molecular subtypes are Luminal A, Luminal B, Her2-enriched and Basal-like. Luminal A and Luminal B are the subtypes with the best prognoses, and are typically treated with hormone therapies. The prognosis is worse for the Her2-enriched subtype, but it has improved with the development of treatments that target receptors of the protein Her2. Finally, the Basal-like subtype has the worst prognosis and currently has no targeted treatment.

Molecular expression varies for each subtype, as shown in [76]. Therefore, characterizing each cancer subtype by its genomic profile can help in its diagnosis and treatment.

#### 4.3.2 Dataset construction

We obtained publicly-available data from The Cancer Genome Atlas (TCGA, https://www.cancer.gov/tcga), using the R package TCGAbiolinks [77]. Specifically, we downloaded gene expression data of patients with breast cancer. This dataset presents the counts of 19,947 genes for 1,215 patients. Gene counts are normalized, which means that the counts were scaled to the total number of reads for each sample. We also downloaded clinical data, including subtype information, which was given for 1,083 patients.

Patients are associated with a total of five subtypes: the ones described above and the ‘Normal-like’ subtype. Due to a low number of examples in our dataset and the fact that this subtype is not always described in articles on breast cancer, we decided to exclude it from our dataset. As the Her2 subtype was also underrepresented, but important, we decided to randomly remove some samples from the other classes to make the dataset more balanced. This makes visualizations easier to interpret in the rest of this section.

Gene expression datasets consist of many genes, but relatively few of them play a role in diseases. To reduce noise in our dataset, we decided to limit the number of features to only keep genes that have been shown to be relevant in breast cancer subtyping. The 50-gene signature described in [78] is widely used to classify breast tumors.

Finally, the dataset was normalized before training.

#### 4.3.3 A3SOM application

As in Section 4.2, we processed the training dataset by removing the labels of an entire class, to manufacture a class that is represented in the data but for which the label is unknown. We chose to remove the labels for the Basal-like subtype as it is the one with the worst prognosis. We also removed at random 40% of labels in the dataset, no matter the class, so that Basal-like was not the only subtype with removed labels. This percentage is chosen based on the complexity of the genomic data and the low number of samples belonging to certain classes (enriched with Her2 in particular): we could remove more labels but some classes can become completely unlabeled or contain very few samples. We then trained the abstained mode of A3SOM to predict the three classes Luminal A, Luminal B and Her2-enriched (denoted LumA, LumB, Her2). Abstention thresholds were set the same way as in the previous section, by looking at mean predictions for each class, and are presented in supplementary file B, Section 3.

**Fig 9:**
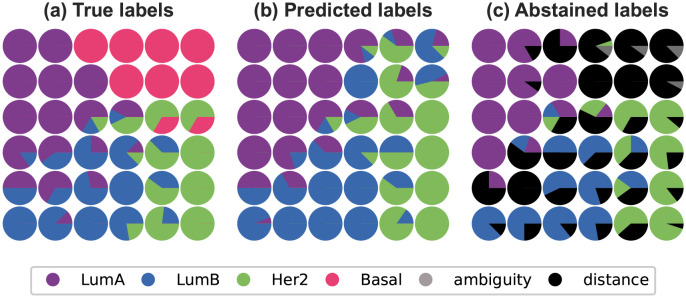
Visualization of the trained SOM with different types of labels for the breast cancer dataset. LumA, LumB and Her2 are the three breast cancer subtypes seen during training, and Basal is the fourth subtype present in the data. Distance and ambiguity are the two abstention criteria.

Fig 9 represents the SOM obtained after training with different information: the true labels associated to samples, the labels predicted by the algorithm, and the labels once abstention has been applied.

Looking at the map in Fig 9c, we can clearly see that the model abstained from predicting an entire group with the distance rule in the top right corner. This group of neurons represents samples that the model identifies as different from the learned classes. Since the neurons are grouped together, the samples they represent are similar. This is a strong indication that these samples might form a new class that was unknown during training. We can compare the labels for these neurons with the true labels on Fig 9a, which confirms that these samples belong to a different class. The model is able to detect a new class in the data. In the context of breast cancer, the true labels (Fig 9a) indicate that these patients all have the aggressive Basal-like subtype. If we do not apply abstention (Fig 9b), the model predicts that these patients have one of the other subtypes. As explained in the biological context, it is important to separate the Basal-like subtype from others, as it is the one with the worst prognosis, and it does not respond to the same therapies as others. Mis-predicting a patient as one of the other subtype when they have Basal breast cancer is dangerous, as it would mean using a treatment that is inappropriate for the patient, potentially experiencing side effects, while letting the tumor grow and become even more aggressive.

#### 4.3.4 Interpretation

We show an example of results interpretation of A3SOM on breast cancer classification. To make the interpretation intelligible, we chose to study the representation of four genes across classes. The genes were selected based on their involvement in breast cancer subtypes [79], but one could replace them by other genes of interest. Indeed, ESR1 (*Estrogen Receptor 1*) and PGR (*Progesterone Receptor*) were chosen for their roles in Luminal subtypes. LumA and LumB are both characterized by high expressions of ER and/or PR genes (respectively Estrogen Receptor and Progesterone Receptor). ERBB2 (*Erb-B2 Receptor Tyrosine Kinase 2*), also called HER2, is the principal gene that characterizes the Her2 subtype. The Basal subtype is sometimes referred to as ‘Triple-negative’ because it has low expression of ER, PR and HER2 markers. Finally, we used MKI67 (*Marker Of Proliferation Ki-67*) to differentiate between LumA and LumB. Based on this, we expect the different signatures described in Table 3.

**Table 3:** Gene signature expected for each subtype. ‘+’ means overexpression of the gene in the subtype, ‘-’ means underexpression, and ‘**/**’ means we have no particular expectation.

	LumA	LumB	Her2	Basal
**ESR1**	+	+	-	-
**PGR**	+	+	-	+
**ERBB2**	-	-	+	**/**
**MKI67**	-	-	-	**/**

**Fig 10:**
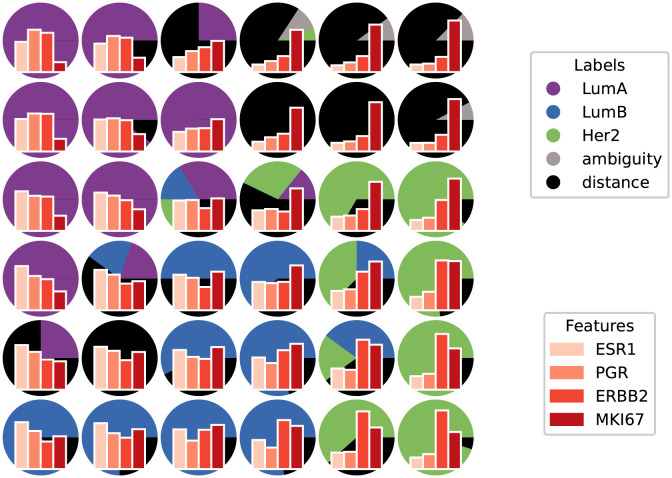
Visualization of the SOM prototypes. Each prototype is represented by its values for four genes. In the background, neurons are also colored by their abstained label, as shown in the previous figure.

Fig 10 shows the SOM with abstained label information in the background, and feature values for the four selected genes in the foreground, for each neuron.

On Fig 10 we see that the expected signatures can be read on prototypes. Prototypes associated with the Her2 subtype, in green, tend to have low values for ESR1 and PGR, and the highest values for ERBB2 compared with other prototypes. Most prototypes associated with the Luminal subtypes have higher values for ESR1 and PGR, and LumB (blue) prototypes have higher expressions of MKI67 than LumA (purple) prototypes. The prototypes in the top-right corner, where labels were primarily rejected with distance abstention, all seem to share a similar signature with low values for the first three genes (ESR1, PGR and ERBB2). It shows that these samples likely belong to the same class, as they have similar signatures, and that they do form a separate group from known classes, as their signatures are quite different. Moreover, this matches the expectations we have for the Basal subtype.

Visualizing the different signatures (feature values) of all subtypes could be a first step to identify biomarkers.

## 5 Discussion

We present in this paper an original method that is, to our knowledge, the only method that can simultaneously handle semi-labeled data, perform abstained classification, provide an explanation for the prediction and produce visualization that helps analyze the model. We showed that our method is competitive to semi-supervised black-box approaches (VIME, TABNET, …) in addition to providing explanation. We detail in the following the comparison between our approach and SOM-based methods.

SuSi [53] was primarily designed to handle hyperspectral data, while A3SOM is generic and can be applied on different applications (A3SOM showed good results on various datasets). Another difference is that although the model does propose classification, so far the authors mainly focused on developing and optimizing their proposal for regression, which can explain why SuSi’s results were relatively low in our classification benchmark (Section 4.1). For BSSSOM (as well as the other variants) [50–52], the base algorithm distinguishes unlabeled and labeled samples during training, to either use an unsupervised algorithm or a supervised algorithm. While the map’s topology stays constant in A3SOM, BSSSOM uses a map with a time-varying structure, to which neurons can be added during training to better represent data. This is a drawback in terms of visualization: as the map is not constrained, the addition of neurons deforms its structure, the notion of neighborhood loses some of its meaning, and it cannot be represented in the same way as A3SOM’s rectangular grid. Conceptually, these three methods diverge in their type of semi-supervision as described in Section 2.1. SuSi performs unsupervised preprocessing. The algorithm is based on two consecutive stages, where the first (unsupervised) task is not modified by the second (supervised) task. BSSSOM also performs two separate learning tasks, either supervised or unsupervised depending on the presence of a label for a sample. As these two tasks happen simultaneously to optimize the model, BSSSOM can be seen as intrinsically semi-supervised. A3SOM is truly intrinsically semi-supervised, as labeled and unlabeled samples are both included in the objective function. Both types of data simultaneously play a role in the optimization of the model’s weights.

Concerning the abstention strategies associated to SOM in the literature, ROSOM [55] and the method in [56] do not distinguish between ambiguity and distance abstention rules which makes the distinction between class discovery and class overlaps difficult. IRSOM [57] only detects ambiguities, but in our case distance is also very important. SLSOM [58] separates the two rules but uses global abstention thresholds which under-performed local thresholds in our experiments. We do not compare our results to other abstained classifiers as abstention options could theoretically be applied after prediction for other methods as well. What makes the originality of A3SOM is that combining the two criteria with the self-organizing map makes abstention decisions interpretable. When we represent abstention results on the SOM, we can understand whether samples for which the distance rule was applied might be outliers or whether they are likely to belong to a new class.

Interpretability has become a necessity in critical areas (e.g., medical, financial, self-driving cars and legal). In the current state-of-the-art, there are two main approaches to interpreting neural networks [80]: post-hoc approaches which explain already trained neural networks and which are the most used, and self-explaining approaches (or explainable) that aim to create interpretable models. Due to the multitude of drawbacks of post-hoc methods, the scientific community recommends building directly interpretable models [81]. Some methods are thus based on examples or prototypes [60] and provide intuitive explanations for the predictions by selecting representative prototypes (or instances) of the input sample, while others provide the most relevant concepts (or features) that lead to the prediction [82]. Our approach follows recent recommendations as it is self-explaining and provides prediction explanation based on prototypes and visualization. In Section 4.3 we have shown how powerful this type of explanation can be. Explainability is not offered by SuSi and BSSSOM. SuSi proposes visualization of the map, but does not address prediction explanation. Due to the varying structure of the map in BSSSOM, visualization is not straightforward, and a prototype-based explanation is not addressed.

## 6 Conclusion

We presented a new approach that combines SOM and dense layers in a semi-supervised context as well as an abstention task. A3SOM can classify data competitively with state-of-the-art algorithms and allows the detection of new classes and class overlaps. In addition to these results, the novelty of our proposal is that using SOM as well as two distinct abstention rules enables the visualization and explanation of classification. The specificity of A3SOM lies in its ability to handle partially labeled data, for which obtaining labels requires many resources. Its use is therefore particularly appropriate in fields where much data is generated, but its study is still in the exploratory stage.

One potential improvement to our model would be to include abstention during the training phase. In the survey [83], three architectures for abstained classification are described: separated, dependent, or integrated rejection. We perform dependent rejection: abstention is applied to the output of the model, after the training step. It would be interesting to explore how integrated rejection affects the model. This would mean applying abstention during training and learning where to apply abstention.

Additional visualizations can be added to develop the model’s explainability further. Examples of visualizations could be the U-matrix, which represents the distance between the neurons of the SOM and shows which neurons form clusters and which are very different, or feature heatmaps [84] to understand the importance of the different features in the definition of the neurons’ prototypes and to identify correlations between features.

One drawback of SOM is that a fixed number of neurons must be defined before training. Several extensions of SOM, called *growing SOM*, have been proposed in the literature [85] to overcome this problem. Since the visualization aspect is very important in our model, a perspective is to extend our model to growing SOM with fixed structure like growing cell structure [86, 87] and growing grid [88, 89].

Future work will further develop this method to study cancer with multi-omics data. Multi-omics, or integrated omics, is the study of multiple molecular levels (genome, transcriptome, proteome…) at the same time. Combining several levels in a computational model is a task that has gained in popularity over the last five years, and has resulted in discoveries in biology. Omics data exist in abundance, but only some are labeled, making semi-supervised approaches relevant. We are interested in making discoveries: it is important that our model can abstain from returning predictions and detect potential new classes. Moreover, bioinformatics is a field rich in interactions between different actors, and providing a visualization to make results easier to understand is also beneficial.

## Supporting information

S1 Text A3SOM source code, as well as supplementary files, are available at https://forge.ibisc.univevry.fr/ccreux/A3SOM.git. (TXT)
